# Supplementary material for: Uncovering intersecting stigmas experienced by people affected by podoconiosis in Nyamasheke district, Rwanda
Source: PLoS Negl Trop Dis. 2024 Oct 21;18(10):e0012603. doi: 10.1371/journal.pntd.0012603 (PMC11527326; doi:10.1371/journal.pntd.0012603)
Supplement: Attachment — Submitted filename: Review comments PNTD-D-24-00874.docx [file pntd.0012603.s001.docx]

**General comments**

This paper addresses an important issue in NTD research. The findings presented can be translated into policy and action not only in the context of podoconiosis prevention and control, but also for other stigmatizing NTDs.

Minor comments

1. Either the short title or the full title needs revision. The two titles seem to convey different meaning. The core theme of the study is "intersectionality" of podoconiosis stigma. I would suggest the title to be revised as "...: Uncovering the intersectionality of podoniosis-related stigma in Rwanda and the short title to read as "Intersectionality of podoconiosis-related stigma..."

2. In the abstract, the last statement in the conclusion section is incomplete. The reader may ask, "To achieve what? What are the implications of this conclusion to the broader scientific audience, policy makers and implementers?"

3. The statement on line 99 is not true. Studies conducted by Tora et al, 2011, 2012 and 2014 and Ayode et al. 2013 and 2016 explored the various manifestations of stigma from social psychological perspective. The authors can articulate their points that "previous studies have not investigated the intersectionality of stigma with social identities".

4. On line 126 and 127, the phrase "identities based sociodemographic ..." is confusing. Revision is needed here.

5. On line 138, the word "illnesses" should be replaced by "stigma".

6. Lines 146-150, needs revision including the relevance of this study to NTD researchers, policy makers and implementers.

7. On line 207, "attitude" is included in the observation checklist. How is it possible to observe attitude?

8. The findings presented demographic characteristics beginning from line 258 should be categorized under an overarching theme. I suggest general themes like 'causes of stigma' or 'sources of stigma'.

9. Why did the authors didn't address the 'intersectionality' of the sources of stigma with social identities such as age, gender, economic status in separate sections?
