# Supplementary material for: Uncovering intersecting stigmas experienced by people affected by podoconiosis in Nyamasheke district, Rwanda
Source: PLoS Negl Trop Dis. 2024 Oct 21;18(10):e0012603. doi: 10.1371/journal.pntd.0012603 (PMC11527326; doi:10.1371/journal.pntd.0012603)
Supplement: Attachment — Submitted filename: Comments_Podo stigma.docx [file pntd.0012603.s002.docx]

**Study title : When you have this disease, and you have no money, nobody can respect you”:**

**Uncovering the understanding of podoconiosis-related stigma in Rwanda.**

**General comments**

It is well designed study with interesting outcomes

However, for publication it is too much detailed, it should be summarized.

After all this intense analysis and discussion , it is good to have clear and elaborated recommendations to alleviate podoconiosis related stigma

**Specific comments**

Page 4, line 82-83 : “out of 23 countries 18 belong to LMIC”, does this mean podocniosis is found in 5 high income countries?

Page 5, line 106 : the term ‘dirtiness’ is highly stigmatizing word and good to reconsider using such word.

Page 7, line 172 : it is stated as ‘ there are 33 individuals belonging to traditional religion”. Do you mean only 33 individuals follow traditional religion in such parts of Africa.

Page 9, line 198 : ‘each participant gave written consent after asking questions and receiving responses’- please rephrase it, as it looks questioning or data collection was conducted before informed consent.

Page 11, line 249-256 : assigning percentage in such small counts (2-15) does not look statistically acceptable
