# Supplementary material for: Uncovering intersecting stigmas experienced by people affected by podoconiosis in Nyamasheke district, Rwanda
Source: PLoS Negl Trop Dis. 2024 Oct 21;18(10):e0012603. doi: 10.1371/journal.pntd.0012603 (PMC11527326; doi:10.1371/journal.pntd.0012603)
Supplement: Attachment — Submitted filename: Responses to the reviewers.docx [file pntd.0012603.s003.docx]

Dr Jean Paul Bikorimana,

Centre for Gender Studies,

University of Rwanda.

Email: igira170@gmail.com

Tel: +250 783566018

20^th^ September 2024

Dear Editor,

**Re: “*When you have this disease, and you have no money, nobody can respect you”:* Uncovering the understanding of podoconiosis-related stigma in Rwanda.**

My co-authors and I would like to resubmit the above-titled revised manuscript for consideration for publication. We have carefully reviewed the comments received and we thank you for your comments and observation. Detailed point-by-point responses to the comments/questions raised are provided below.

Thank you for consideration of our manuscript, which we hope is now suitable for publication.

We look forward to hearing from you.

Yours sincerely

Dr Jean Paul Bikorimana

Dear Reviewers,

Thank you very much for your constructive comments on our manuscript. Your comments are of great help to us in improving our manuscript. We have read your comments and made corrections in accordance with your suggestions and comments. Details of how corrections and revisions have been made are as following:

| **Reviewer 1** | **Responses** |
| --- | --- |
| Either the short title or the full title needs revision. The two titles seem to convey different meaning. The core theme of the study is "intersectionality" of podoconiosis stigma. I would suggest the title to be revised as "...: Uncovering the intersectionality of podoconiosis-related stigma in Rwanda and the short title to read as "Intersectionality of podoconiosis-related stigma..." | We thank the reviewer for commenting on the title, and I appreciate the suggestion. We originally avoided using the term ‘intersectionality’ in the title because of other manuscripts we are working. However, we agree that the title is better revised, and it now reads “**Uncovering intersecting stigmas experienced by people affected by podoconiosis in Nyamasheke district, Rwanda”. For a small title, it reads “Experience of Intersecting stigmas related to podoconiosis.** [See the title page] |
| In the abstract, the last statement in the conclusion section is incomplete. The reader may ask, "To achieve what? What are the implications of this conclusion to the broader scientific audience, policy makers and implementers?" | Many thanks for this very important comment. The overall contribution and achievement of our study is that we have demonstrated that the experience of podoconiosis related stigma is intersectional, beyond the biological aspects of the disease. For the broader scientific audience, our research offers new insights into how stigma operates across neglected tropical diseases. For policy makers and implementers, it underscores the importance of integrating intersectionality into public health strategies and policies aimed at reducing stigma and promoting social inclusion. We have added these implications and hope they will be satisfactory. See p 2. Lines 52 -59 |
| The statement on line 99 is not true. Studies conducted by Tora et al, 2011, 2012 and 2014 and Ayode et al. 2013 and 2016 explored the various manifestations of stigma from social psychological perspective. The authors can articulate their points that "previous studies have not investigated the intersectionality of stigma with social identities". | Thanks for this comment. We agree with the comment. Kindly note that we have revised it as you have suggested. See p. 4-5 Line 113 - 125 |
| On line 126 and 127, the phrase "identities based sociodemographic ..." is confusing. Revision is needed here. | Kindly, note that the revision has been made. The term socio-economic class was used in the manuscript. See p. 6 line 150 |
| On line 138, the word "illnesses" should be replaced by "stigma". | Thank you for the suggestion, and kindly note that the replacement has been made. See p. 6 line 162 |
| Lines 146-150, needs revision including the relevance of this study to NTD researchers, policy makers and implementers. | Many thanks for the comment. We have altered this paragraph to read “By using intersectionality, our findings shed light on ways in which multiple aspects such as biological features of podoconiosis, social identities, and structures intersect to shape the experience of podoconiosis stigma. Scholars have demonstrated that NTDs are associated with factors beyond illness that are stigmatising.  This emphasises the importance of understanding the nature of podoconiosis stigma, and other NTDs by investigating how the intersection of multiple social identities, systems and the aspects of illness contribute to the stigma. Therefore, our study can inform policies and the development of inclusive interventions aimed at reducing podoconiosis stigma that target not only podoconiosis features, but also pay attention to individual identities and social systems that contribute to the experience of stigma.”  See p. 7 lines 175 –184 |
| On line 207, "attitude" is included in the observation checklist. How is it possible to observe attitude? | Thanks to the reviewer for this comment. While observing attitudes directly would be challenging, through conversations certain non-verbal cues such as facial expressions, body gestures and interactions between individuals may signal negative or positive attitudes. We have changed this to read “behaviours and practices”. See p. 10 line 245 |
| The findings presented demographic characteristics beginning from line 258 should be categorized under an overarching theme. I suggest general themes like 'causes of stigma' or 'sources of stigma'. | The overarching theme for this paper is the source of stigma related to podoconiosis, and this was broken down into three major sources of stigma. We include demographic information to give the readers an overview of the study participants and context. However, following the comment, we have included an introductory statement to direct readers through the findings section. See p. 12 on lines 284- 288 |
| Why did the authors didn't address the 'intersectionality' of the sources of stigma with social identities such as age, gender, economic status in separate sections? | Many thanks to the reviewer for asking this very interesting question. There are many ways of structuring and presenting our findings. Given that the purpose of this paper was to argue that podoconiosis experience is intersectional, we prefer to discuss how more than one identity may intersect with biological features of illness. For example, we discussed how disease severity and economic class worked together to influence the experience of stigma related to physical appearance of foot and legs. This would not be possible if we were to present individual social identities separately. |
| Reviewer 2 |  |
| While the list of factors with some evidence of relevance to stigma is comprehensive, I think explanatory models (around causality) are particularly key and could be expanded upon a little. These of course intersect with different characteristics of individuals and groups, reinforcing stereotypes mentioned. Religion and different approaches to health care (traditional vs modern/western) are mentioned, and this would be of key interest, not only because of different ideas of causality that populations might have in relation to the impact on stigma, but also because it would affect design of both stigma interventions, and help-seeking behavior in general. | Many thanks for this comment. We agree that explanatory models around podoconiosis are linked to stigma and play a big role in help seeking. In our study, we identified various explanatory models for podoconiosis, including terms used to describe podoconiosis, causality [poison, witchcraft, walking in forbidden places, hereditary], symptoms, onset, treatment etc. While some of the explanatory models were not linked to stigma, terms and causality were associated with stigma, which varied from individual to individual due to various identities and social category they belonged to. Discussion of the link between explanatory models of podoconiosis and stigma, and intersectionality has been added in the manuscript for your consideration. Kindly see p. 16, line 376 -382. P 17 line 411-413  While we concur with the reviewer about the role explanatory models play in shaping the healthcare seeking journey, healthcare seeking behaviours were not central to this paper. |
| The methods, sampling etc are very appropriate for the study, and it makes sense for the study to be carried out in an area with high podoconiosis prevalence. Sampling is well described (though some more detail around what constituted a 'perceived' patient would be useful). | The concept of ‘perceived’ patients was used in this study to gain a deeper understanding of how individuals affected by podoconiosis were perceived in the community. During interviews with patients, they were asked whether they knew someone else with the same condition and to describe the person physically. Afterwards, questions were asked about podoconiosis symptoms. These explanations have been added into the manuscript and we hope that the revision is satisfactory. Kindly See p. 9. Lines 221-225 . |
| Ethical approvals are well described and overall in the paper there is a welcome sense of how the global cooperation worked. It is useful to go beyond the description of ethical approvals/consent process, to also explore what some of the ethical issues that might be expected would be, and describe how these would be addressed in the methods and engagement with participants. | Many thanks to the reviewer for raising this point. We wish we could discuss ethics-related experiences and issues, but this would form another research paper on its own. The idea of discussing ethics issues such as power dynamics between researchers and participants, emotional trauma experienced from listening to the painful stories, and dilemmas related to securing a real informed consent to participate in the study are worth discussing in a separate manuscript. The ethics section in this manuscript is simply to show that our study was approved by relevant ethics committees and that we followed required ethical procedures. We have added a very brief description of some of the ethical issues experienced a the end of this section. See p. 11-12 line 280-282. |
| While the Author's Summary gives a good lay description of the study, I think there would still be value in explaining the meaning of words like intersectionality and positionality when first used. This very brief primer would help some readers have a better insight into why these are important. While recognising that the research referenced was probably carried out prior to the name change, I suggest you add Eswatini/eSwatini in parentheses after the mention of Swaziland. | According to your suggestion, brief definitions of intersectionality and positionality have been added to the manuscript. See p. 3 line 102 – 103 for positionality, and p. 4 line 113-117 for intersectionality.  We have also added ‘Eswatini’ in brackets after ‘Swaziland’. P 5 139 |
| The results are comprehensive and rich, and it is good to see the reference to where intersections occur, which is laid out in a good level of detail. In general they are in keeping with established findings, but add some valuable detail, that would be of value to implementers in this area. | Thanks to the reviewer for appreciation, it is our hope that the information included in this paper would be of useful for the readers and implementers, particularly in this area.  Discussion of how the findings of this study might inform policy and action has been added in the manuscript. See p 15-16 line 366-374; p. 18 line 434 -444; p. 23 line 548-558  Through the planned dissemination workshops, we hope to discuss the relevant issues in more detail with implementers and stakeholders in the area. |
| The use of quotes is effective and adds colour to the results. Please translate 'ibidido' (line 305, page 13), which I assume means 'podoconiosis' but may contain more subtle meaning that would be useful for a non-Kinyarwanda speaker to understand. | Thanks for the comment. *Ibidido* can be literally translated as greatly swollen feet and legs. This meaning carries a pejorative meaning and it is associated with stigma. Kindly note that the translation has been added. See p. 14 on line 351 |
| The results described are followed through well in the discussion, but I wonder whether a table or graphic of some sort might make the learning more accessible and systematised. The figure on page 26 is useful, but quite high level, and some more details around how the identities and stigmatising factors intersect might be possible to represent graphically. This is an idea, and may or may not be considered appropriate or useful by the authors. | Thanks for this concern. Visualising the intersectionality is complex, and can be presented in various ways. In the figure, we tried to highlight various factors at different levels and explained how they intersect to shape the podoconiosis related experience in the study area. Apologies, we have not come up with a better way of representing the intersection of identities and stigmatizing factors. |
| Under 'limitations', the authors correctly refer to the results being contextual. It would be helpful to hear their thoughts on either how the actual learning from this area might general elsewhere, or how the methods and approach might be applied elsewhere so that this research and methodology can inform work in other locations. | This is a great comment and thank to the reviewer to rise this point of generalization. We have slightly expanded this paragraph as follows:  “The study’s findings are specific to the context in which the research was conducted, which includes particular cultural, socio-economic, and geographical factors. As such, the findings may not be directly transferable to other settings with different social dynamics and health challenges. Also, the observation time was short, and this may have prevented us from observing more realities in relation to the experience of stigma. Future studies may consider conducting similar studies in in different geographic and cultural settings to have a broader understanding of stigma operates across various environments. These studies might consider longer observation times, which would help in unpacking more nuanced realties about the nature and dynamics of podoconiosis related stigma.”  Kindly find these revisions on P 30 line 700 – 710.  . |
| Reviewer 3 |  |
| Page 4, line 82-83 :  “out of 23 countries 18 belong to LMIC”, does this mean podoconiosis is found in 5 high income countries? | We have revised these figures as we realized they were not correct. The most recent study on the global distribution of podoconiosis found evidence of presence of podoconiosis in 17 countries, 12 were African countries, two Asian countries and 3 from Latin America. For the revision made, see p. 4 on line 93 – 95 |
| Page 5, line 106: the term ‘dirtiness’ is highly stigmatizing word and good to reconsider using such word. | Kindly note that your comment has been considered and the term dirtiness has been replaced by inadequate hygiene. See P. 5 on line 129, and p.14 on line 342. |
| Page 7, line 172: it is stated as ‘there are 33 individuals belonging to traditional religion”. Do you mean only 33 individuals follow traditional religion in such parts of Africa. | Many thanks for the question, and this number is for the study area alone, not country wide, so additional information has been added in the manuscript for clarity. See p. 8 on line 206 -207 |
| Page 9, line 198: ‘each participant gave written consent after asking questions and receiving responses’ - please rephrase it, as it looks questioning or data collection was conducted before informed consent. | Kindly note that the revision has been made to the manuscript. See p. 10 line 236 - 237 |
| Page 11, line 249-256 : assigning  percentage in such small counts (2-15) does not look statistically acceptable | Thanks for this comment – we have removed the percentages. Kindly see p. 12 line 290 – 302 |
